# Trajectories of lipid profile with cognitive function: 12-year follow-up of Guangzhou Biobank cohort study

**DOI:** 10.1007/s00406-025-01974-5

**Published:** 2025-02-28

**Authors:** Yu Meng Tian, Wei Sen Zhang, Chao Qiang Jiang, Feng Zhu, Ya Li Jin, Shiu Lun Au Yeung, Jiao Wang, Kar Keung Cheng, Tai Hing Lam, Lin Xu

**Affiliations:** 1 School of Public Health, Sun Yat-Sen University, 74 Zhongshan 2nd Road, Guangzhou, China; 2 Guangzhou Twelfth People’s Hospital, Guangzhou, China; 3 School of Public Health, The University of Hong Kong, Hong Kong, , China; 4 Department of Applied Health Sciences, University of Birmingham, Birmingham, UK; 5 Greater Bay Area Public Health Research Collaboration, Guangzhou, China

**Keywords:** Lipid profiles, Cholesterol, Memory function, Cognition, Longitudinal

## Abstract

Studies on the trajectories of lipid profile with cognitive decline are scarce and inconsistent. We examined this association and explored potential effect modification. Lipid profiles including high-density lipoprotein cholesterol (HDL-C), low-density lipoprotein cholesterol (LDL-C), triglycerides (TG) and total cholesterol (TC) were measured at baseline, first and second follow-ups, and classified into trajectory groups. Immediate, delayed memory recall and cognitive function were assessed by the immediate (IWRT) and delayed 10-word recall test (DWRT), and mini-mental state examination (MMSE), respectively, at baseline and follow-ups, and converted into standardized scores. Linear mixed-effect model was used. Results show that elevated HDL-C was associated with increasing annual change rates in DWRT and MMSE scores (β (95% confidence intervals) = 0.005 (-0.001 to 0.011) SD/year and 0.020 (0.014 to 0.027) SD/year, respectively), while LDL-C and TC were associated with a decrease in DWRT scores (-0.005 (-0.008 to -0.001) SD/year and − 0.009 (-0.011 to -0.006) SD/year, respectively). Moderate-increasing HDL-C, high-decreasing LDL-C or moderate-decreasing TC trajectories were associated with increases in DWRT, IWRT and MMSE scores. Stronger associations were identified for HDL-C with an increase in MMSE scores within the lower family income group and for LDL-C with a decrease in IWRT scores among individuals taking lipid-lowering drug. In conclusion, HDL-C was associated with improvements, while LDL-C and TC with decline in memory and cognitive function. Increasing HDL-C and decreasing LDL-C and TC trajectories predicted better memory and cognitive performance. The observed effect modifications highlight the importance of personalized approaches in lipid management to optimize cognitive outcomes.

**Supplementary Information:**

The online version contains supplementary material available at 10.1007/s00406-025-01974-5.

## Introduction

Due to the rapid population aging, Alzheimer’s disease (AD) has become a great public health challenge, characterized by a set of symptoms such as memory loss and impaired thinking abilities [1]. Notably, effective treatment for AD is unknown to date. Since cognitive decline is considered as the preclinical symptom of AD [2], early identification of early interventions against the antecedent conditions or risk factors of cognitive decline particularly in older adults are needed which may delay the onset and development of AD [3].

Abnormal lipid metabolism has been implicated in the pathogenesis of AD [4]. However, the association between lipids and cognitive decline is complex and multifactorial. Previous studies found that higher midlife lipid levels measured at a single time point, were associated with an increased risk of cognitive decline [5, 6]. As single-time-point measurements of lipids fail to capture their dynamic changes over time, particularly in older adults [7], examining the long-term trajectories of lipid levels is crucial for a comprehensive understanding of their associations with cognitive decline. Several studies have examined the association between lipid profile trajectories and cognitive decline, but the findings were inconsistent [8–13]. For example, some studies focused on either total cholesterol (TC) [9–12] or low-density lipoprotein cholesterol (LDL-C) [13], reporting negative [10–12], positive [13] or null [9] associations with cognitive decline. Only one study examining changes in all lipid parameters based on six time points from childhood to midlife showed positive associations for increasing TC and LDL-C trajectories, but no associations for high-density lipoprotein cholesterol (HDL-C) and triglycerides (TG) trajectories, with memory decline [8]. To date, we found no report on the association between changes in lipid profiles during middle to older age and cognitive decline. Furthermore, most previous studies conducted in the US or other developed countries, where hyperlipidemia and lipid-lowering drug use are more prevalent [14]. A recent meta-analysis of 21 cohort studies showed that statin use was significantly associated with a lower risk of AD [15]. Nearly half of Americans used statins [16], and failing to account for this may lead to an underestimation of the association between lipid profiles and cognitive decline. Therefore, exploring this association in a population with a low prevalence of hyperlipidemia (< 15%) and statin use (< 5.0%) [17], such as the Chinese population, might provide less confounded results.

Therefore, we examined the prospective association of changes in lipid profiles with cognitive decline in middle-aged and older Chinese using data from the Guangzhou Biobank Cohort Study, taking into account various potential confounding factors as well as lipid-lowering drugs. We also explored what factors could modify the relationships.

## Methods

### Study sample and setting

The Guangzhou Medical Ethics Committee of the Chinese Medical Association approved the study and all participants gave written, informed consent before participation. Details of the Guangzhou Biobank Cohort Study (GBCS) have been reported previously [18–20]. Briefly, the GBCS is a three-way collaboration among Guangzhou Twelfth People’s Hospital and the Universities of Hong Kong and Birmingham. Participants were drawn from the Guangzhou Health and Happiness Association for the Respectable Elders (GHHARE), from September 2003 to January 2008. The GHHARE was unofficially aligned with municipal government and had branches in all districts of Guangzhou, the capital city of Guangdong Province in Southern China. Membership of the GHHARE was open to Guangzhou permanent residents aged 50 years or above for a nominal fee of 4 CNY (≈ 50 US cents) per month. About 7% of Guangzhou residents in this age group were included in the GHHARE.

All surviving participants were invited to return for the first (March 2008 to December 2012) and second follow-up examinations (March 2013 to December 2017). Both baseline and follow-up examinations included a face-to-face computer-assisted interview by trained nurses to collect information on demographic characteristics, lifestyle factors, and family and personal medical history. Participants fasted overnight before coming to the examination center early in the morning, blood was taken at about 9 a.m. Anthropometric and clinical parameters such as fasting plasma glucose, blood pressure, lipids and inflammatory markers were measured. The follow-up questionnaire and clinical and laboratory examinations were mostly similar to those at baseline. The reliability of the questionnaire was tested by randomly recalling 200 participants for re-interview and the results were satisfactory [18].

### Lipid profiles measurement

Lipid profiles including HDL-C, LDL-C, TG, apolipoprotein A1 (apoA1), apolipoprotein B (apoB) and TC in mmol/L were measured by Roche COBAS automatic biochemical analyzer in the clinical laboratory of the Guangzhou Twelfth People’s Hospital [18]. Among them, HDL-C, LDL-C, TG and TC were measured at baseline (2003–2008), first (2008–2012) and second (2012–2020) follow-up examinations, but apoA1 and apoB were measured at baseline only due to resource constraints. Furthermore, patterns of changes in lipid profiles were classified according to trajectory analysis. The use of drug was assessed by the question of ‘Have you taken drug for hypertension, glucose or lipids regularly in the past month?’.

### Cognitive function assessment

Immediate 10-word recall test (IWRT), delayed 10-word recall test (DWRT) and mini-mental state examination (MMSE) were used to assess the immediate, delayed memory recall and cognitive function, respectively, at both baseline (2003–2008), first (2008–2012) and second (2012–2020) follow-up examinations, as reported in our previous GBCS papers [19]. Greater scores indicated better function, and reduction in scores with time indicated decline in function. DWRT or IWRT was a test of verbal learning and memory requiring recall a list of ten words. To better fit Chinese culture, the adapted 10-word list included ‘letter,’ ‘ticket,’ ‘grass,’ ‘arm,’ ‘corner,’ ‘stick,’ ‘book,’ ‘stone,’ ‘chairman,’ and ‘soy sauce’. During the interview, these 10 words were read out to participants one by one. Then the participants were immediately asked to recall the words. Participants were given one point for each word that they could be correctly recalled. This process was repeated three times and summed scores for these three recalls were IWRT (scores from 0 to 30). After five minutes of answering other questions for distraction, participants were asked to recall as many words as they could remember. The last recall was DWRT (scores from 0 to 10). The total number of words was denoted by IWRT and DWRT scores, respectively. Memory impairment was defined by DWRT scores of less than 4, corresponding to one standard deviation (SD) below the mean (mean ± SD: 5.5 ± 1.8) [19]. MMSE was consisted of 11 items (scores from 0 to 30), as reported in our previous GBCS paper [21]. Poor cognitive function was defined by MMSE scores of less than 25, corresponding to one SD below the mean (mean ± SD: 27.5 ± 2.6) [21]. For interpretation and comparison of cognitive decline across different tests, the raw scores were converted to standardized scores according to the baseline scores by subtracting the mean and dividing by the SD [22].

### Potential confounders

Baseline sociodemographic, lifestyle and biological factors, self-rated health, and self-reported history of disease and medication were analyzed as potential confounders. The sociodemographic factors included sex, age, education (junior middle or below, senior middle or above), occupation (manual, non-manual, others), marital status (married, others), and family income (< 29999 CNY/year, 30000–49999 CNY/year, ≥ 50000 CNY/year, unknown; US$1 = 6.95 CNY). Lifestyle factors included physical activity (inactive, minimally active, active), smoking status (never, ever) and drinking status (never, ever), and biological factors included body mass index (BMI). Physical activity was assessed by the International Physical Activity Questionnaire (IPAQ), which had been validated in our previous paper [20]. Self-reported health status (poor/very poor), history of disease and medication including cardiovascular disease, hypertension, diabetes, hyperlipidemia and drug for hypertension, glucose or lipids, was also assessed by experienced nurses in the interviews.

### Statistical analysis

We used the semiparametric group-based trajectory model (GBTM) to identify the potential subgroups of participants (STATA command ‘traj’). The GBTM, a specialized application of finite mixture modeling, was employed to identify groups of participants based on similar developmental trajectories of lipid profile throughout the follow-up [23]. This method assumes population heterogeneity and the existence of a finite number of distinct groups [23]. Model fit was assessed by the Bayesian Information Criterion (BIC) and Average Posterior Probability (AvePP) [24], with a lower BIC and AvePP > 0.7 indicating goodness of fit (Supplementary Table 1). Baseline characteristics by HDL-C trajectory groups were compared by one-way analysis of variance (ANOVA) for continuous variables and chi-squared test for categorical variables. We used the linear mixed-effect model, which is specifically designed for repeated measurements data, with random intercept and slope to explore the longitudinal association of lipid profiles and lipid profile trajectory groups with changes in standardized DWRT, IWRT and MMSE scores over time, respectively, yielding regression coefficients (βs) and 95% confidence intervals (CIs). In accordance with previous studies, the interaction terms of lipid profiles/lipid profile trajectory groups × follow-up time (years) were added to the model, respectively. The estimate for interaction indicates the extent of longitudinal association between lipid profile levels or lipid profile trajectory groups and the annual change rates in standardized scores (SD per year) [22]. We also tested the interactions between lipid profiles and potential effect modifiers including sex, age (60/≥60 years), education (≤ junior/≥senior middle school), family income (30000/≥30000 CNY/year) and lipid-lowering drug (no/yes) by adding the three-way interaction items (i.e., lipid profiles × follow-up time × sex/age/education/family income/lipid-lowering drug). Regarding the adjustment for confounders, we included two model. Model 1 was adjusted for baseline sociodemographic, lifestyle and biological factors and self-rated health. Model 2 was additionally adjusted for self-reported history of disease and medication. Furthermore, to be consistent with previous studies, we also used multivariable linear regression to analyze the longitudinal associations of baseline lipid profiles and lipid profile trajectory groups with the mean annual change in scores, yielding βs and 95% CIs. Mean annual change in scores was calculated by the change in standardized scores (the second follow-up standardized scores minus baseline standardized scores) divided by follow-up time in years. All data analysis was performed using STATA/SE 16.0 (Stata Corp LP, College Station, TX, USA), and the two-sided p value < 0.05 was considered statistically significant.

## Results

Of 8,847 participants with all variables of interest, after excluding those with memory impairment or poor cognitive function at baseline (*N* = 840), 8,007 participants were included in the current analysis (Supplementary Fig. 1). Of them, 3,314 participants also had MMSE. The average age of participants at baseline was 59.4 years (SD = 6.1), and 74% were women. The average follow-up duration was 8.1 years (SD = 1.5).

**Fig. 1:**
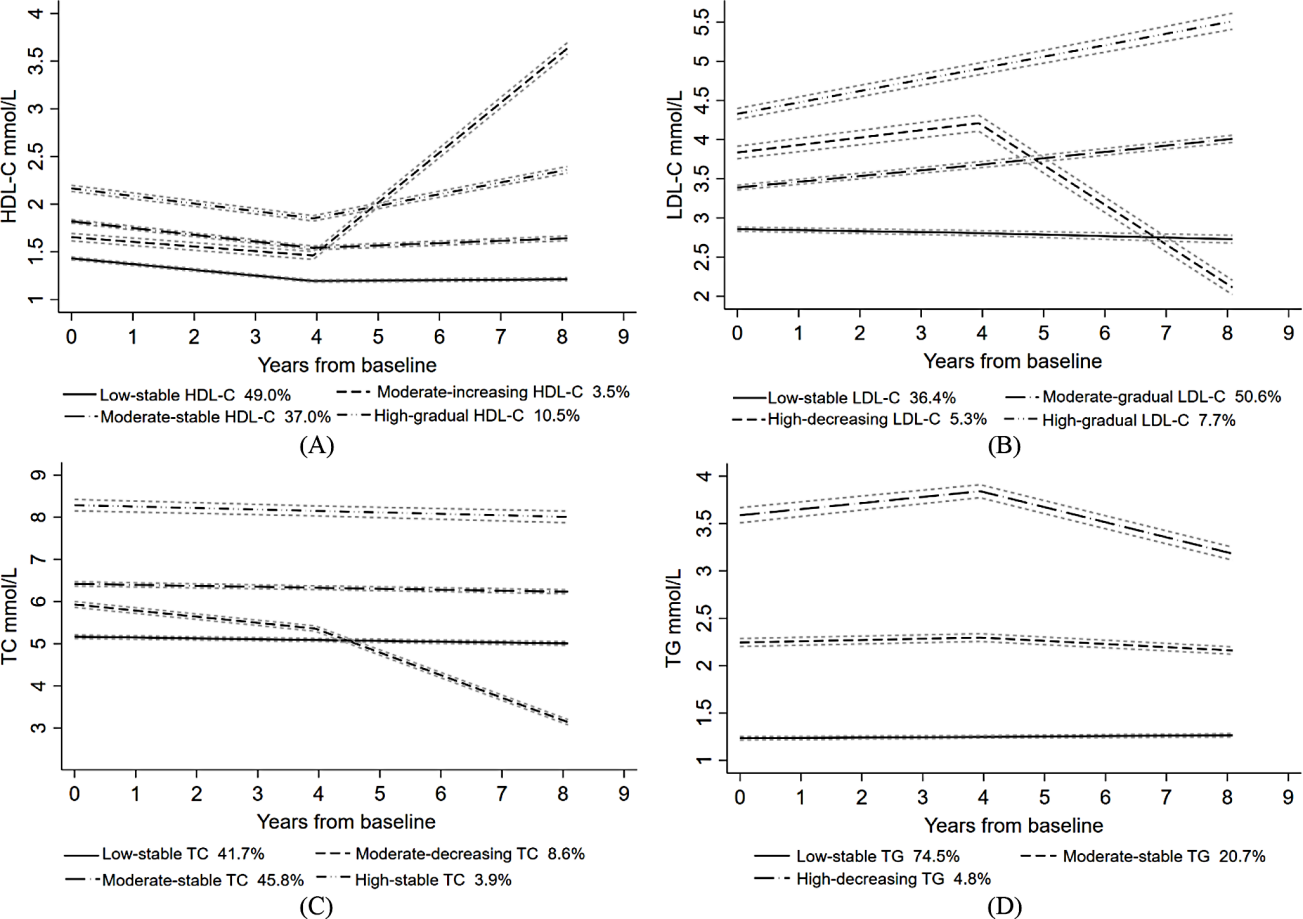
HDL-C (**A**), LDL-C (**B**), TC (**C**) and TG (**D**) trajectory groups during 12-year follow-up Note: (1) Values are means and 95% confidence interval (CI) for each serum lipid. (2) Four trajectory groups for HDL-C (**A**), LDL-C (**B**) and TC (**C**), and three trajectory groups for TG (**D**) were identified by group-based trajectory model. (3) HDL-C = high-density lipoprotein cholesterol; LDL-C = low-density lipoprotein cholesterol; TC = Total cholesterol; TG = Triglycerides

In Fig. 1, HDL-C, LDL-C and TC were optimally classified into four trajectory groups and TG into three trajectory groups. HDL-C included low-stable (49.0%), moderate-stable (37.0%), high-gradual (10.5%) and moderate-increasing (3.5%) groups. LDL-C included low-stable (36.4%), moderate-gradual (50.6%), high-gradual (7.7%) and high-decreasing (5.3%) groups. TC included low-stable (41.7%), moderate-stable (45.8%), high-stable (3.9%) and moderate-decreasing (8.6%) groups. TG included low-stable (94.9%) and high-increase (5.1%) trajectory groups. TG included low-stable (74.5%), moderate-stable (20.7%) and high-decreasing (4.8%) groups.

Table 1 shows that at baseline, compared to participants with low-stable HDL-C, those with moderate-increasing HDL-C had a greater proportion of women, were younger, had a greater proportion of being manual workers and ever drinkers, married and physically active, and higher prevalence of hyperlipidemia. They also had a lower proportion of ever smokers, lower level of education and BMI, lower prevalence of cardiovascular disease, hypertension, diabetes and lower proportion of taking drug for hypertension, glucose and lipids (all *p* < 0.05). Similar patterns were found in participants with high-gradual HDL-C, except for the lower proportion of being married and ever drinkers, higher level of education and lower prevalence of hyperlipidemia. No association of HDL-C trajectory groups with family income and self-rate health was found (*p *from 0.17 to 0.46).

**Table 1:** Baseline characteristics of participants by HDL-C trajectory group, Guangzhou Biobank Cohort Study, 2003–2008

	HDL-C trajectory groups				*p* value^*^
	Low-stable	Moderate-stable	High-gradual	Moderate-increasing	
N (%)	3926 (49.0)	2960 (37.0)	839 (10.5)	282 (3.5)	
Sex, N (%)					
Women	2557 (65.1)	2440 (82.4)	717 (85.5)	229 (81.2)	< 0.001
Men	1369 (34.9)	520 (17.6)	122 (14.5)	53 (18.8)	
Age, mean (SD), years	59.7 (6.0)	59.4 (6.2)	58.4 (6.2)	57.7 (5.6)	< 0.001
Education, N (%)					
Junior middle or below	2370 (60.4)	1791 (60.6)	457 (54.5)	172 (61.0)	0.01
Senior middle or above	1556 (39.6)	1167 (39.5)	382 (45.5)	110 (39.0)	
Occupation, N (%)					
Manual	2164 (55.5)	1693 (57.6)	464 (55.8)	171 (61.5)	0.01
Non-manual	1115 (28.6)	745 (25.4)	213 (25.6)	59 (21.2)	
Others	618 (15.9)	500 (17.0)	154 (18.5)	48 (17.3)	
Marital status, N (%)					
Married	2295 (87.6)	1754 (85.3)	465 (82.9)	190 (89.6)	< 0.001
Others	326 (12.4)	303 (14.7)	96 (17.1)	22 (10.4)	
Smoking status, N (%)					
Never	3115 (79.4)	2623 (88.8)	761 (90.9)	251 (89.0)	< 0.001
Ever	808 (20.6)	332 (11.2)	76 (9.1)	31 (11.0)	
Drinking status, N (%)					
Never	2591 (66.3)	2110 (71.6)	586 (70.5)	184 (65.5)	< 0.001
Ever	1317 (33.7)	836 (28.4)	245 (29.5)	97 (34.5)	
Family income, CNY/year, N (%)					
< 29,999	1315 (33.5)	991 (33.5)	299 (35.6)	86 (30.6)	0.46
30,000–49,999	1006 (25.6)	758 (25.6)	228 (27.2)	81 (28.8)	
≥ 50,000	873 (22.3)	643 (21.7)	178 (21.2)	68 (24.2)	
Unknown	729 (18.6)	567 (19.2)	134 (16.0)	46 (16.4)	
Physical activity					
Inactive	360 (9.2)	209 (7.1)	85 (10.1)	23 (8.2)	< 0.001
Minimally active	1563 (39.8)	1081 (36.5)	277 (33.0)	105 (37.2)	
Active	2003 (51.0)	1670 (56.4)	477 (56.9)	154 (54.6)	
BMI, mean (SD), kg/m^2^	24.4 (3.0)	23.2 (3.1)	22.0 (3.2)	23.6 (2.8)	< 0.001
Self-rated health, poor/very poor, N (%)	552 (14.4)	447 (15.5)	141 (17.3)	38 (14.1)	0.17
Cardiovascular disease, yes, N (%)	1649 (42.1)	1055 (35.7)	285 (34.0)	103 (36.8)	< 0.001
Hyperlipidemia, yes, N (%)	528 (13.5)	261 (8.8)	79 (9.4)	39 (13.8)	< 0.001
Diabetes, yes, N (%)	298 (7.6)	131 (4.4)	25 (3.0)	12 (4.3)	< 0.001
Hypertension, yes, N (%)	1075 (27.4)	627 (21.2)	141 (16.8)	59 (21.0)	< 0.001
Drug for hypertension, glucose or lipids, yes, N (%)	1212 (32.9)	702 (25.0)	155 (19.6)	68 (25.5)	< 0.001

HDL-C = high-density lipoprotein cholesterol; N = number of participants; SD = standard deviation; BMI = body mass index

US$1 = 6.95 CNY

^*^: The *p* values < 0.05 indicated significant differences across the HDL-C trajectory groups

Table 2, model 2 shows that after adjusting for potential confounders, HDL-C was significantly associated with increasing annual change rates (increase) in MMSE scores (adjusted β (95% CI) = 0.020 (0.014 to 0.027) SD/year), whereas the association with DWRT scores was not statistically significant (0.005 (-0.001 to 0.011) SD/year). No association between HDL-C and annual change rates in IWRT scores was found (-0.003 (-0.009 to 0.003) SD/year, *p* = 0.29). LDL-C was significantly associated with decreases in DWRT (-0.005 (-0.008 to -0.001) SD/year) and MMSE scores (-0.008 (-0.012 to -0.004) SD/year). TC was significantly associated with decreases in DWRT and IWRT scores (-0.009 (-0.011 to -0.006) SD/year and − 0.005 (-0.007 to -0.003) SD/year, respectively), but not MMSE scores (-0.002 (-0.004 to 0.001) SD/year, *p* = 0.20). No association of TG with annual change rates in DWRT, IWRT and MMSE scores was found (*p* from 0.32 to 0.93). Supplementary Table 2 shows that no association of baseline apoA1 and apoB levels with mean annual change in DWRT, IWRT and MMSE scores was found using multivariable linear regression.

**Table 2:** Longitudinal association of lipid profiles with the annual change rates in standardized DWRT scores, IWRT scores and MMSE scores based on linear mixed-effect model during 12-year follow-up

	Crude β (95% CI)	*p* value	Model 1^a^	*p* value	Model 2^b^	*p* value
**DWRT**						
HDL-C × time^#^	-0.006 (-0.011, -0.001)^*^	0.04	0.005 (-0.001, 0.011)	0.12	0.005 (-0.001, 0.011)	0.12
LDL-C × time^#^	-0.009 (-0.013, -0.006)^***^	< 0.001	-0.004 (-0.008, -0.001)^*^	0.02	-0.005 (-0.008, -0.001)^*^	0.02
TC × time^#^	-0.008 (-0.010, -0.006)^***^	< 0.001	-0.008 (-0.011, -0.006)^***^	< 0.001	-0.009 (-0.011, -0.006)^***^	< 0.001
TG × time^#^	-0.001 (-0.005, 0.003)	0.49	-0.002 (-0.007, 0.002)	0.32	-0.002 (-0.007, 0.002)	0.32
**IWRT**						
HDL-C × time^#^	-0.010 (-0.015, -0.005)^***^	< 0.001	-0.003 (-0.009, 0.003)	0.30	-0.003 (-0.009, 0.003)	0.29
LDL-C × time^#^	-0.002 (-0.005, 0.001)	0.21	0.002 (-0.001, 0.006)	0.19	0.002 (-0.001, 0.006)	0.22
TC × time^#^	-0.005 (-0.007, -0.003)^***^	< 0.001	-0.005 (-0.007, -0.003)^***^	< 0.001	-0.005 (-0.007, -0.003)^***^	< 0.001
TG × time^#^	0.001 (-0.002, 0.005)	0.52	0.001 (-0.004, 0.005)	0.78	0.001 (-0.004, 0.005)	0.76
**MMSE**						
HDL-C × time^#^	0.024 (0.017, 0.031)^***^	< 0.001	0.020 (0.014, 0.027)^***^	< 0.001	0.020 (0.014, 0.027)^***^	< 0.001
LDL-C × time^#^	-0.008 (-0.013, -0.004)^***^	< 0.001	-0.008 (-0.012, -0.004)^***^	< 0.001	-0.008 (-0.012, -0.004)^***^	< 0.001
TC × time^#^	-0.003 (-0,005, -0.001)^*^	0.04	-0.002 (-0.004, 0.001)	0.19	-0.002 (-0.004, 0.001)	0.20
TG × time^#^	0.001 (-0.004, 0.006)	0.82	-0.001 (-0.005, 0.005)	0.96	-0.001 (-0.005, 0.005)	0.93

DWRT = delayed 10-word recall test; IWRT = immediate 10-word recall test; MMSE = mini-mental state examination; HDL-C = high-density lipoprotein cholesterol; LDL-C = low-density lipoprotein cholesterol; TG = triglycerides; TC = total cholesterol; CI = confidence interval

^a^: Model 1: adjusted for sex, age, baseline standardized IWRT/DWRT/MMSE scores, education, occupation, marital status, smoking status, drinking status, family income, physical activity, BMI, and self-rated health

^b^: Model 2: additionally adjusted for self-reported cardiovascular disease, hyperlipidemia, diabetes, hypertension, and drug for hypertension, glucose or lipids

^#^: β coefficient and its 95% CI were reported as SD per year

^*^: *p* < 0.05; ^**^: *p* < 0.01; ^***^: *p* < 0.001

Table 3, model 2 shows that, after similar adjustment as above, compared with participants with low-stable HDL-C, those with moderate-increasing HDL-C showed significantly increasing annual change rates (increase) in DWRT, IWRT and MMSE scores (adjusted β (95% CI) = 0.044 (0.024 to 0.064) SD/year, 0.028 (0.009 to 0.047) SD/year and 0.025 (0.009 to 0.042) SD/year, respectively). DWRT and IWRT scores, but not MMSE scores, significantly increased in those with high-decreasing LDL-C (0.039 (0.021 to 0.057) SD/year, 0.027 (0.010 to 0.045) SD/year and 0.008 (-0.008 to 0.025) SD/year, respectively). Significant increase in DWRT and MMSE scores, but not IWRT scores were found in those with moderate-decreasing TC (0.038 (0.025 to 0.052) SD/year, 0.020 (0.007 to 0.034) SD/year and 0.013 (-0.001 to 0.026) SD/year, respectively). No association between TG trajectory groups and annual change rates in DWRT, IWRT and MMSE scores was found (*p *from 0.33 to 0.80). In addition, consistent results on the associations of lipids profile trajectory groups with the mean annual change in DWRT, IWRT and MMSE scores were found using multivariable linear regression (Supplementary Table 3).

**Table 3:** Longitudinal association of lipid profile trajectory groups with the annual change rates in standardized DWRT scores, IWRT scores and MMSE scores based on linear mixed-effect model during 12-year follow-up

	Crude β (95% CI)	*p* value	Model 1^a^	*p* value	Model 2^b^	*p* value
**DWRT**						
HDL-C trajectory groups × time^#^, N (%)						
Low-stable, 3926 (49.0)	0.000		0.000		0.000	
Moderate-stable, 2960 (37.0)	-0.005 (0.011, 0.002)	0.14	-0.004 (-0.013, 0.004)	0.31	-0.004 (-0.013, 0.004)	0.30
High-gradual, 839 (10.5)	0.009 (-0.001, 0.019)	0.09	0.012 (-0.001, 0.025)	0.08	0.012 (-0.001, 0.025)	0.08
Moderate-increasing, 282 (3.5)	0.034 (0.019, 0.049)^***^	< 0.001	0.044 (0.024, 0.063)^***^	< 0.001	0.044 (0.024, 0.064)^***^	< 0.001
LDL-C trajectory groups × time^#^, N (%)						
Low-stable, 2900 (36.4)	0.000		0.000		0.000	
Moderate-gradual, 4037 (50.6)	0.001 (-0.006, 0.007)	0.81	0.004 (-0.004, 0.012)	0.32	0.004 (-0.004, 0.012)	0.33
High-gradual, 612 (7.7)	-0.003 (-0.016, 0.009)	0.59	-0.002 (-0.018, 0.013)	0.76	-0.003 (-0.018, 0.013)	0.75
High-decreasing, 419 (5.3)	0.024 (0.011, 0.037)^***^	< 0.001	0.039 (0.021, 0.057)^***^	< 0.001	0.039 (0.021, 0.057)^***^	< 0.001
TC trajectory groups × time^#^, N (%)						
Low-stable, 3319 (41.7)	0.000		0.000		0.000	
Moderate-stable, 3657 (45.8)	0.006 (-0.001, 0.012)	0.07	0.006 (-0.002, 0.014)	0.17	0.006 (-0.003, 0.014)	0.17
High-stable, 307 (3.9)	0.002 (-0.014, 0.018)	0.81	0.001 (-0.019, 0.021)	0.94	0.001 (-0.019, 0.021)	0.95
Moderate-decreasing, 674 (8.6)	0.036 (0.026, 0.046)^***^	< 0.001	0.038 (0.024, 0.052)^***^	< 0.001	0.038 (0.025, 0.052)^***^	< 0.001
TG trajectory groups × time^#^, N (%)						
Low-stable, 5804 (74.5)	0.000		0.000		0.000	
Moderate-stable, 1614 (20.7)	-0.003 (-0.010, 0.005)	0.50	-0.005 (-0.015, 0.005)	0.33	-0.005 (-0.015, 0.005)	0.33
High-decreasing, 371 (4.8)	0.001 (-0.014, 0.014)	0.99	-0.008 (-0.027, 0.010)	0.38	-0.008 (-0.027, 0.010)	0.38
**IWRT**						
HDL-C trajectory groups × time^#^, N (%)						
Low-stable, 3926 (49.0)	0.000		0.000		0.000	
Moderate-stable, 2960 (37.0)	0.003 (-0.003, 0.008)	0.40	0.001 (-0.008, 0.008)	0.95	0.001 (-0.008, 0.008)	0.97
High-gradual, 839 (10.5)	0.005 (-0.004, 0.014)	0.28	0.007 (-0.006, 0.020)	0.31	0.006 (-0.007, 0.019)	0.35
Moderate-increasing, 282 (3.5)	0.024 (0.010, 0.038)^*^	0.01	0.028 (0.009, 0.047)^**^	0.004	0.028 (0.009, 0.047)^**^	0.003
LDL-C trajectory groups × time^#^, N (%)						
Low-stable, 2900 (36.4)	0.000		0.000		0.000	
Moderate-gradual, 4037 (50.6)	0.004 (-0.0002, 0.010)	0.20	0.006 (-0.002, 0.014)	0.15	0.006 (-0.002, 0.014)	0.16
High-gradual, 612 (7.7)	0.005 (-0.006, 0.017)	0.37	0.010 (-0.005, 0.025)	0.19	0.010 (-0.005, 0.025)	0.20
High-decreasing, 419 (5.3)	0.016 (0.004, 0.028)^*^	0.01	0.027 (0.010, 0.045)^**^	0.002	0.027 (0.010, 0.045)^**^	0.002
TC trajectory groups × time^#^, N (%)						
Low-stable, 3319 (41.7)	0.000		0.000		0.000	
Moderate-stable, 3657 (45.8)	0.004 (-0.002, 0.010)	0.18	0.004 (-0.004, 0.012)	0.37	0.003 (-0.005, 0.011)	0.40
High-stable, 307 (3.9)	-0.001 (-0.015, 0.014)	0.96	-0.001 (-0.020, 0.018)	0.91	-0.001 (-0.020, 0.018)	0.91
Moderate-decreasing, 674 (8.6)	0.011 (0.002, 0.020)^*^	0.02	0.013 (-0.001, 0.026)	0.06	0.013 (-0.001, 0.026)	0.06
TG trajectory groups × time^#^, N (%)						
Low-stable, 5804 (74.5)	0.000		0.000		0.000	
Moderate-stable, 1614 (20.7)	-0.003 (-0.010, 0.003)	0.32	-0.002 (-0.012, 0.007)	0.63	-0.002 (-0.012, 0.007)	0.64
High-decreasing, 371 (4.8)	0.005 (-0.008, 0.018)	0.41	0.006 (-0.012, 0.024)	0.51	0.006 (-0.012, 0.024)	0.51
**MMSE**						
HDL-C trajectory groups × time^#^, N (%)						
Low-stable, 1509 (47.9)	0.000		0.000		0.000	
Moderate-stable, 1064 (33.8)	0.006 (-0.003, 0.015)	0.18	0.007 (-0.003, 0.016)	0.17	0.007 (-0.003, 0.016)	0.17
High-gradual, 395 (12.6)	0.002 (-0.010, 0.015)	0.70	0.004 (-0.009, 0.017)	0.58	0.004 (-0.009, 0.017)	0.51
Moderate-increasing, 179 (5.7)	0.023 (0.008, 0.038)^**^	0.002	0.025 (0.008, 0.042)^**^	0.003	0.025 (0.009, 0.042)^**^	0.002
LDL-C trajectory groups × time^#^, N (%)						
Low-stable, 1060 (33.7)	0.000		0.000		0.000	
Moderate-gradual, 1533 (48.7)	-0.005 (-0.014, 0.003)	0.23	-0.006 (-0.016, 0.003)	0.19	-0.006 (-0.015, 0.003)	0.21
High-gradual, 329 (10.5)	-0.006 (-0.020, 0.008)	0.42	-0.007 (-0.022, 0.008)	0.35	-0.007 (-0.022, 0.008)	0.36
High-decreasing, 222 (7.1)	0.009 (-0.006, 0.024)	0.22	0.008 (-0.008, 0.024)	0.32	0.008 (-0.008, 0.025)	0.31
TC trajectory groups × time^#^, N (%)						
Low-stable, 1294 (41.1)	0.000		0.000		0.000	
Moderate-stable, 1402 (44.6)	0.001 (-0.008, 0.009)	0.95	-0.002 (-0.011, 0.007)	0.68	-0.002 (-0.011, 0.007)	0.72
High-stable, 126 (4.0)	-0.001 (-0.021, 0.020)	0.99	-0.001 (-0.023, 0.021)	0.92	-0.001 (-0.023, 0.021)	0.92
Moderate-decreasing, 325 (10.3)	0.021 (0.009, 0.033)^**^	0.001	0.020 (0.007, 0.033)^**^	0.003	0.020 (0.007, 0.034)^**^	0.003
TG trajectory groups × time^#^, N (%)						
Low-stable, 2327 (75.6)	0.000		0.000		0.000	
Moderate-stable, 626 (20.3)	-0.001 (-0.011, 0.009)	0.90	-0.002 (-0.012, 0.009)	0.76	-0.002 (-0.012, 0.009)	0.76
High-decreasing, 127 (4.1)	-0.006 (-0.026, 0.014)	0.54	-0.003 (-0.024, 0.019)	0.81	-0.003 (-0.024, 0.018)	0.80

DWRT = delayed 10-word recall test; IWRT = immediate 10-word recall test; MMSE = mini-mental state examination; HDL-C = high-density lipoprotein cholesterol; LDL-C = low-density lipoprotein cholesterol; TG = triglycerides; TC = total cholesterol; CI = confidence interval; N = number of participants

^a^: Model 1: adjusted for sex, age, baseline standardized IWRT/DWRT/MMSE scores, education, occupation, marital status, smoking status, drinking status, family income, physical activity, BMI, and self-rated health

^b^: Model 2: additionally adjusted for self-reported cardiovascular disease, hyperlipidemia, diabetes, hypertension, and drug for hypertension, glucose or lipids

^#^: β coefficient and its 95% CI were reported as SD per year

^*^: *p* < 0.05; ^**^: *p* < 0.01; ^***^: *p* < 0.001

**Fig. 2:**
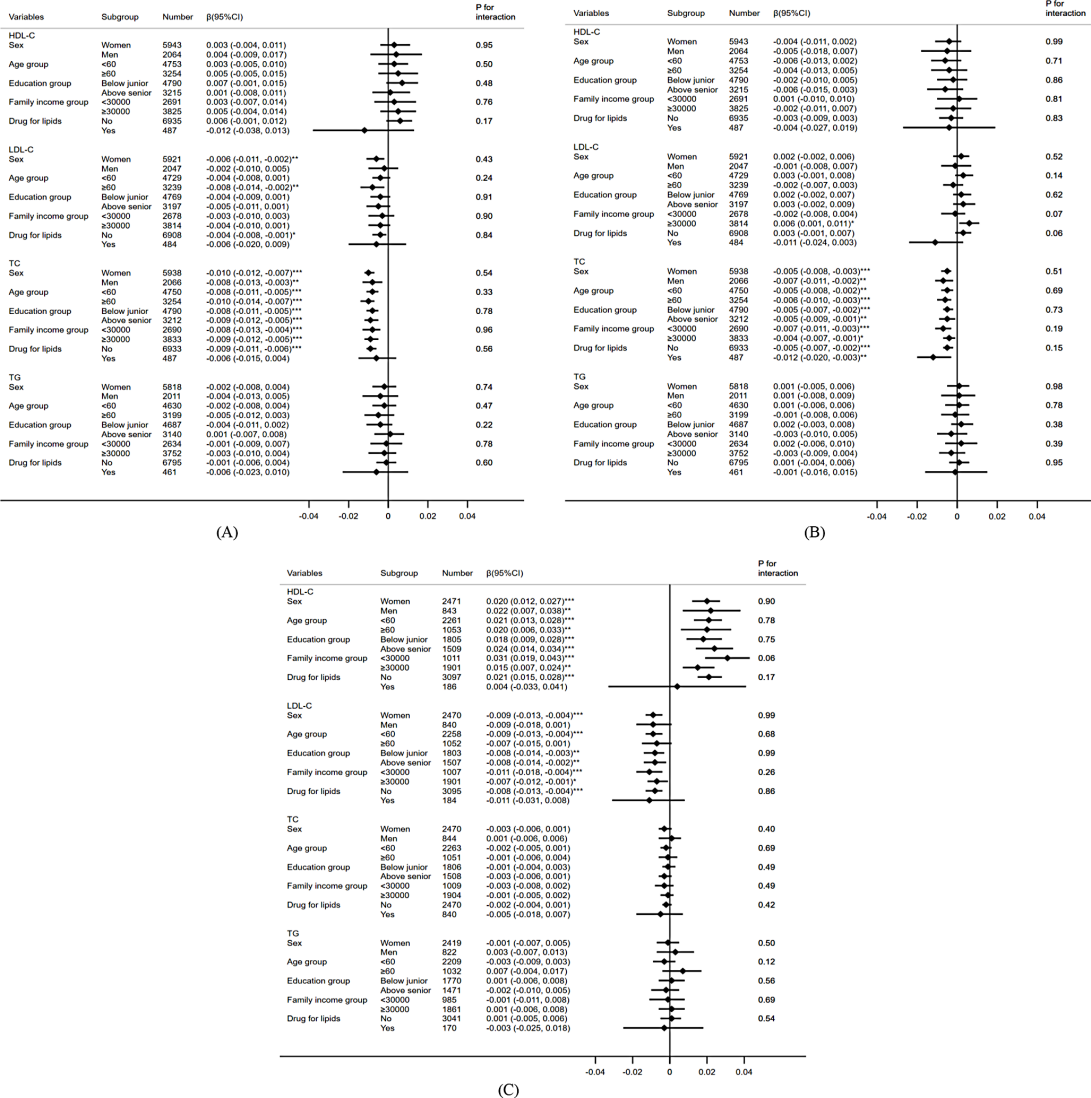
Forest plots for the association between lipid profiles and the annual change rates in standardized DWRT (**A**), IWRT (**B**) and MMSE scores (**C**) stratified by sex, age group, education group, family income group and lipid lowering drug group based on linear mixed-effect model during 12-year follow-up. Note: (1) β and 95% CI were adjusted for sex, age, baseline standardized IWRT/DWRT/MMSE scores, BMI, education, occupation, marital status, smoking status, drinking status, family income, physical activity, self-rated health, self-reported cardiovascular disease, hypertension, diabetes, hyperlipidemia and drug for hypertension, glucose or lipids. (2) β = coefficient; CI = confidence interval; Number = number of participants; DWRT = delayed 10-word recall test; IWRT = immediate 10-word recall test; MMSE = mini-mental state examination; HDL-C = high-density lipoprotein cholesterol; LDL-C = low-density lipoprotein cholesterol; TC = total cholesterol; TG = triglycerides

Figure 2 shows that the associations of lipid profiles with the annual change rates in standardized DWRT, IWRT and MMSE scores did not vary by sex, age and education (*p* for interactions from 0.12 to 0.99). However, participants with lower family income appeared to have stronger association of HDL-C with increasing annual change rates (increase) in MMSE scores than those with higher family income (0.031 (0.019 to 0.043) SD/year and 0.015 (0.007 to 0.024) SD/year, respectively, *p* for family income interaction = 0.06). Additionally, LDL-C was non-significantly associated with a decrease in IWRT scores in participants with lipid-lowering drug (-0.011 (-0.024 to 0.003) SD/year), while associated with an increase in IWRT scores in those without lipid-lowering drug (0.003 (-0.001 to 0.007) SD/year, *p* for lipid-lowering drug interaction = 0.06).

## Discussion

### Principal findings

Our study is the first report of the largest cohort study spanning 12 years from middle to older age that, after accounting for potential confounders and lipid-lowering drugs comprehensively, HDL-C was significantly associated with increase, while LDL-C and TC with decrease in DWRT and MMSE score, with higher scores indicating better cognitive function. No association of TG with changes in memory and cognitive function was found. Additionally, compared to participants with the low-stable status of lipid profiles, those with increasing HDL-C, decreasing LDL-C or decreasing TC trajectories predicted greater improvements in memory and cognitive function. The interaction results show a stronger association of HDL-C with an increase in MMSE scores in participants with lower family income than those with higher family income, and an association of LDL-C with a decrease in IWRT scores in participants taking lipid-lowering drug.

### Comparison with previous studies

#### Association of HDL-C with memory and cognitive improvements

Our results of a positive association of elevated HDL-C with the annual changes in memory function and cognitive function were consistent with some [25–27] but not at all [6, 28, 29] previous studies, which all had smaller sample size than the present study. A longitudinal study of 1,114 Japanese with a mean age of 73.5 years and a follow-up of 19 years showed that higher HDL-C levels in midlife were associated with a lower risk of late-life mild cognitive impairment (MCI), a condition considered an early stage in the progression to AD [25]. However, cognitive function was only assessed at the end of follow-up. Another longitudinal study of 774 Swedish aged over 50 years also found a positive association of HDL-C with cognitive function in women after 10 years follow up [26]. The other cohort study of only 98 Spanish aged over 60 years with both lipid profiles and cognitive function measured at four different time points during 1-year follow-up showed that elevated HDL-C was associated with better cognitive performance [27], but the rate of cognitive decline was not assessed. Furthermore, two longitudinal studies based on China Health and Retirement Longitudinal Study (CHARLS) of Chinese aged over 45 years with a 4-year follow-up, one study of 4,915 participants with lipid profiles and cognitive function measured at two time points [29] and the other one of 6,792 participants with lipid profiles measured at baseline and cognitive function measured at two time points [6], found no association of HDL-C with risk of cognitive decline. The non-significant association was also reported by another longitudinal study of 1,159 older Chinese with a mean age of 80 years and a follow-up of 5 years [28]. Notably, due to the occurrence of faster cognitive decline commonly observed in older adults, the potentially protective effect of HDL-C, if any, could be less apparent or unclear and difficult to detect. Additionally, the highly heterogeneous particle size of HDL-C might also contribute to the inconsistent results. A recent cohort study of 1,991 participants aged over 61 years in Hong Kong examining the association of different HDL-C particle subspecies with incident cardiovascular disease and all-cause mortality showed a negative association for small HDL-C but a positive association for very large HDL-C during a 5-year follow-up [30]. Hence, future studies to clarify the relationships between different HDL-C particle subspecies and changes in cognitive function are warranted.

#### Association of TC and LDL-C with memory and cognitive decline

Both elevated TC and LDL-C were significantly associated with memory and cognitive decline in our study, similar to findings from previous studies that only measured lipid profiles at baseline [31, 32]. For instance, a longitudinal study of 2,514 Chinese with a median age of 59 years and an average follow-up of 2.3 years showed that higher baseline TC and LDL-C levels were associated with accelerated cognitive decline [32]. Another cohort study of 7,103 Americans aged over 65 years measuring TC at baseline and cognitive function at three time points during the 10-year follow-up showed that higher baseline TC levels was associated with greater cognitive decline [31]. However, some studies found either null [33] or inverse [10, 34] association of TC and LDL-C levels with cognitive decline. For example, a longitudinal study of 2,675 Americans with a mean age of 50 years and a 5-year follow-up found non-significant association of baseline TC levels with the risk of cognitive decline [33]. In this study, cognitive decline was defined as greater than 1.5 SD from the mean change in the cognitive scores, which might misclassify mild decline to no change. In addition, treating cognitive decline as a dichotomize variable might reduce the statistical power and also ignore people with improved scores. Two other cohort studies, one of 1,604 Americans aged over 38 years with a 32-year follow-up [34] and the other of 1,462 Swiss aged over 50 years with a 25-year follow-up [10], reported that lower baseline TC levels were associated with a higher risk of AD. The inverse association between TC and higher risk of AD might be explained by olfactory dysfunction rather than TC per se [35]. The deposition of amyloid protein as the prodrome of AD resulted in a reduction in the volume of the primary olfactory cortex, thereby leading to olfactory impairment [36], which could subsequently reduce food intake and lower plasma TC levels. Nonetheless, our study primarily focused on cognitive and memory decline as the preclinical indicators of AD, and thus the issue of reverse causation (i.e., AD → olfactory impairment → reduced TC) might be minimized. Hence, our results, together with the smaller studies mentioned above, suggest that LDL-C and TC might be casually related to decline in cognitive function.

#### Effect modification of family income and lipid-lowering drug

Furthermore, our results indicated potential interactions of lipid profiles with sex, age and education on cognitive function, suggesting women, older and taking lipid-lowering drug participants experienced greater cognitive decline which was associated with elevated LDL-C and TC levels, but participants with higher education levels and lower family income were less susceptible, which was consistent with some previous studies [28, 31, 32, 37]. Regarding the moderator of family income, stronger association of HDL-C with better cognitive function in adults with lower family income was found, which has not been reported in previous studies. This might be because those with lower family income generally had higher proportion of manual occupation (i.e., farmers and workers) than those with higher family income, and might have much lower levels of meat intake.

Additionally, our study observed a non-significant trend indicating that LDL-C was associated with a decrease in IWRT scores in participants on lipid-lowering drugs, whereas participants not taking these drugs exhibited an increase in IWRT scores. This observation aligns with findings from previous cohort studies, suggesting that lipid-lowering drugs, such as statins, may influence cognitive performance, particularly in working memory. For example, a large cohort study of 184,367 US adults over a 2-year follow-up found a positive association between baseline LDL-C levels and risk of AD in statin users, while an inverse association was observed in nonusers [37]. Similarly, another longitudinal study from the UK Biobank indicated that statin use was associated with a reduction in working memory [38]. One proposed explanation is that lipid-lowering drugs may disrupt cholesterol homeostasis, potentially leading to a cholesterol deficit in the brain. Such a deficit could affect the myelination process, which is critical for efficient information transmission and cognitive function, particularly in memory [38]. However, despite the larger sample size of our study than previous studies, it was plausible that the statistical power might have been inadequate to detect effect modification. Subsequent research with greater numbers of participants is needed to analyzed effect modification of the above associations.

### Mechanisms

There are some possible mechanisms underlying the associations between unfavorable lipid profiles and cognitive decline. For instance, the cholesterol levels within the blood-brain barrier of neuronal membrane lipid rafts increase concurrently. This increase, in turn, could augment the enzymatic activity of β-site amyloid precursor protein cleavage enzyme-1 (BACE-1) and γ-secretase. This heightened enzymatic activity accelerates the cleavage of amyloid precursor protein (APP), resulting in elevated production of β-amyloid plaques, a key pathological feature of AD [4]. These plaques are neurotoxic, disrupting synaptic function and potentially leading to cognitive decline. Conversely, HDL-C may play a protective role in cognitive health. Evidence suggests that HDL-C can reduce the accumulation of β-amyloid plaques and mitigate endothelial inflammation, both of which may contribute to slower cognitive decline [39]. Furthermore, genetic factors, such as the APOE gene, might also modulate these relationships [40]. The APOE gene encodes a critical component of HDL particles involved in lipid transport and metabolism and may involve in the development of AD [40]. The presence of certain AOPE alleles may interact with lipid profiles to influence cognitive trajectories, underscoring the complexity of the mechanisms linking lipids to cognitive outcomes.

### Limitations

Several limitations should be acknowledged in the present study. First, cognitive assessment in the GBCS focused on memory and global cognitive function, without inclusion of other cognitive domains such as executive function and language. Further investigations with comprehensive and systematic evaluation of cognitive function are warranted. Second, due to the exclusion of participants lacking follow-up cognitive assessments, the presence of selection bias was unavoidable. It was possible that participants experiencing accelerated cognitive decline or diagnosed with AD might have been unable to attend the follow-up examinations, potentially leading to underestimation of the strength of the associations. Third, due to the lack of information on important genetic variants related to cognitive function (i.e., APOE gene), we could not assess the gene-environment interactions in this study. However, previous studies suggested that the frequency of E4 allele was significantly lower in Chinese people than in other regions such as South Asians and Europeans [41], thus the results might not be substantially biased. Fourth, the GBCS participants might not be representative of the general older population in China. They might have less fluctuation of lipid profiles and thus the associations could be more difficult to be observed. Fifth, the non-significant association of baseline apoA1 and apoB levels with mean annual change in memory and cognitive function might be due to the small number of participants with apoA1 and apoB measured. However, a recent randomized controlled trial also showed no association of baseline apoA1 and other subclass of HDL-C with the risk of AD [42]. Further longitudinal studies clarifying the association of changes in apoA1 and apoB with cognitive function are needed.

## Conclusions

In a comprehensive longitudinal study of a large cohort spanning middle to older ages with substantial follow-up, we found an association between higher levels of HDL-C and improvements in memory and cognitive function. Conversely, higher levels of LDL-C and TC were associated with declines in these functions. No significant association of TG with changes in memory and cognitive function was found. Increasing HDL-C and decreasing LDL-C and TC trajectories predicted better memory and cognitive performance. The observed effect modifications highlight the importance of personalized approaches in lipid management, considering socioeconomic status and medication use, to optimize cognitive outcomes.

## Electronic supplementary material

Below is the link to the electronic supplementary material.

Supplementary Material 1

## Data Availability

Ethical approval in place allows us to share data on requests. Please directly send such requests to the Guangzhou Biobank Cohort Study Data Access Committee (gbcsdata@hku.hk).

## Declarations

### Use of AI and AI-assisted technologies Statement

The authors declare that AI has not been used in the writing process.
