# Supplementary material for: Trajectories of lipid profile with cognitive function: 12-year follow-up of Guangzhou Biobank cohort study
Source: Eur Arch Psychiatry Clin Neurosci. 2025 Feb 28;276(1):291–303. doi: 10.1007/s00406-025-01974-5 (PMC12904904; doi:10.1007/s00406-025-01974-5)
Supplement: Supplementary file 1 — Supplementary Material 1 [file 406_2025_1974_MOESM1_ESM.docx]

**Supplementary Table 1.** Group-based trajectory model results of the fitting process

|  | Number of groups | Log-Lik | BIC | Participants per group (%) | Mean posterior probabilities |
| --- | --- | --- | --- | --- | --- |
| HDL-C | 1 | -17415.54 | 17433.52 | 100 | 1.00 |
|  | 2 | -13633.04 | 13677.98 | 85.4/14.6 | 0.97/0.90 |
|  | 3 | -11932.11 | 11995.03 | 71.0/24.4/4.6 | 0.94/0.88/0.95 |
|  | 4 | -11336.43 | 11421.82 | 49.0/37.0/10.5/3.5 | 0.97/0.80/0.85/0.96 |
| LDL-C | 1 | -31796.23 | 31818.69 | 100 | 1.00 |
|  | 2 | -29676.62 | 29712.55 | 64.0/36.0 | 0.92/0.87 |
|  | 3 | -28808.82 | 28858.22 | 49.7/44.2/6.1 | 0.86/0.88/0.87 |
|  | 4 | -28289.02 | 28378.85 | 50.6/36.4/7.7/5.3 | 0.86/0.86/0.87/0.80 |
| TG | 1 | -28700.29 | 28718.22 | 100 | 1.00 |
|  | 2 | -24828.80 | 24864.66 | 85.2/14.8 | 0.98/0.93 |
|  | 3 | -23886.08 | 23935.39 | 74.5/20.7/4.8 | 0.95/0.87/0.93 |
| TC | 1 | -39901.34 | 39923.81 | 100 | 1.00 |
|  | 2 | -37964.02 | 38008.96 | 51.1/48.9 | 0.87/0.87 |
|  | 3 | -36485.72 | 36544.14 | 60.9/29.9/9.2 | 0.90/0.87/0.95 |
|  | 4 | -35873.33 | 35931.75 | 45.8/41.7/8.6/3.9 | 0.86/0.84/0.93/0.89 |

Log-Lik: the maximum Log-Likelihood; BIC: Bayesian Information Criterion; HDL-C = high-density lipoprotein cholesterol; LDL-C = low-density lipoprotein cholesterol; TG = triglycerides; TC = total cholesterol

**Supplementary Table 2.** Association of baseline lipid profiles with mean annual changes in standardized DWRT scores, IWRT scores, and MMSE scores based on multivariable linear regression during 12-year follow-up

|  | Crude β (95% CI) per SD | Model 1^a^ | Model 2^b^ |
| --- | --- | --- | --- |
| **DWRT** | | | |
| HDL-C | -0.006 (-0.012, 0.001) | -0.007 (-0.015, 0.001) | -0.007 (-0.015, 0.002) |
| LDL-C | -0.001 (-0.005, 0.003) | 0.003 (-0.001, 0.007) | 0.003 (-0.002, 0.007) |
| TG | -0.001 (-0.004, 0.001) | -0.001 (-0.004, 0.002) | -0.001 (-0.004, 0.001) |
| apoA1 | 0.003 (-0.030, 0.036) | -0.007 (-0.037, 0.023) | -0.007 (-0.038, 0.023) |
| apoB | -0.005 (-0.023, 0.012) | -0.005 (-0.020, 0.010) | -0.005 (-0.020, 0.010) |
| apoA1/apoB | -0.002 (0.010, 0.007) | 0.001 (-0.006, 0.008) | 0.001 (-0.006, 0.008) |
| TC | -0.001 (-0.003, 0.002) | -0.001 (-0.004, 0.002) | -0.001 (-0.004, 0.001) |
| **IWRT** | | | |
| HDL-C | 0.001 (-0.006, 0.007) | -0.002 (-0.010, 0.005) | -0.002 (-0.010, 0.005) |
| LDL-C | 0.005 (0.002, 0.009)^***^ | 0.008 (0.004, 0.012)^***^ | 0.008 (0.004, 0.012)^***^ |
| TG | 0.001 (-0.002, 0.002) | 0.001 (-0.002, 0.003) | 0.001 (-0.002, 0.003) |
| apoA1 | 0.024 (-0.007, 0.055) | 0.011 (-0.018, 0.040) | 0.013 (-0.016, 0.042) |
| apoB | -0.014 (-0.030, 0.003) | -0.004 (-0.017, 0.011) | -0.003 (-0.017, 0.011) |
| apoA1/apoB | 0.004 (-0.003, 0.012) | 0.003 (-0.003, 0.010) | 0.004 (-0.003, 0.010) |
| TC | -0.001 (-0.003, 0.002) | 0.001 (-0.002, 0.003) | 0.001 (-0.002, 0.003) |
| **MMSE** | | | |
| HDL-C | 0.003 (-0.005, 0.012) | 0.001 (-0.006, 0.009) | 0.002 (-0.006, 0.010) |
| LDL-C | 0.001 (-0.004, 0.006) | 0.001 (-0.003, 0.006) | 0.001 (-0.003, 0.005) |
| TG | 0.001 (-0.003, 0.003) | 0.001 (-0.001, 0.004) | 0.001 (-0.001, 0.003) |
| apoA1 | 0.001 (-0.020, 0.023) | -0.006 (-0.026, 0.013) | -0.006 (-0.025, 0.014) |
| apoB | 0.010 (-0.013, 0.034) | 0.003 (-0.017, 0.024) | 0.003 (-0.018, 0.023) |
| apoA1/apoB | -0.005 (-0.018, 0.008) | 0.001 (-0.009, 0.011) | 0.001 (-0.009, 0.012) |
| TC | 0.001 (-0.003, 0.003) | 0.001 (-0.002, 0.003) | 0.001 (-0.002, 0.003) |

DWRT = delayed 10-word recall test; IWRT = immediate 10-word recall test; MMSE = mini-mental state examination; HDL-C = high-density lipoprotein cholesterol; LDL-C = low-density lipoprotein cholesterol; TG = triglycerides; apoA1 = apolipoprotein A1; apoB = apolipoprotein B; TC = total cholesterol; CI = confidence interval

^a^: Model 1: adjusted for sex, age, baseline standardized IWRT/DWRT/MMSE scores, BMI, education, occupation, marital status, smoking status, drinking status, family income, physical activity, and self-rated health

^b^: Model 2: additionally adjusted for self-reported cardiovascular disease, hypertension, diabetes, hyperlipidemia and drug for hypertension, glucose or lipids

^*^: P < 0.05; ^**^: P < 0.01; ^***^: P < 0.001**Supplementary Table 3.** Association of lipid profile trajectory groups with mean annual changes in standardized DWRT scores, IWRT scores, and MMSE scores based on multivariable linear regression during 12-year follow-up

|  | Crude β (95% CI) per SD | Model 1^a^ | Model 2^b^ |
| --- | --- | --- | --- |
| **DWRT** | | | |
| HDL-C, N (%) |  |  |  |
| Low-stable, 3926 (49.0) | 0.000 | 0.000 | 0.000 |
| Moderate-stable, 2960 (37.0) | -0.005 (-0.011, 0.002) | -0.001 (-0.008, 0.005) | -0.001 (-0.008, 0.006) |
| High-gradual, 839 (10.5) | 0.007 (-0.003, 0.016) | 0.009 (-0.002, 0.020) | 0.009 (-0.001, 0.020) |
| Moderate-increasing, 282 (3.5) | 0.028 (0.012, 0.044)^**^ | 0.038 (0.022, 0.054)^***^ | 0.039 (0.022, 0.055)^***^ |
| LDL-C, N (%) |  |  |  |
| Low-stable, 2900 (36.4) | 0.000 | 0.000 | 0.000 |
| Moderate-gradual, 4037 (50.6) | 0.005 (-0.001, 0.011) | 0.001 (-0.006, 0.008) | 0.001 (-0.005, 0.008) |
| High-gradual, 612 (7.7) | -0.003 (-0.014, 0.009) | -0.001 (-0.012, 0.011) | -0.001 (-0.012, 0.011) |
| High-decreasing, 419 (5.3) | 0.021 (0.007, 0.034)^**^ | 0.031 (0.017, 0.046)^***^ | 0.032 (0.017, 0.046)^***^ |
| TG, N (%) |  |  |  |
| Low-stable, 5804 (74.5) | 0.000 | 0.000 | 0.000 |
| Moderate-stable, 1614 (20.7) | -0.005 (-0.012, 0.002) | -0.005 (-0.013, 0.003) | -0.006 (-0.013, 0.002) |
| High-decreasing, 371 (4.8) | 0.001 (-0.013, 0.015) | -0.011 (-0.026, 0.004) | -0.012 (-0.027, 0.003) |
| TC, N (%) |  |  |  |
| Low-stable, 3319 (41.7) | 0.000 | 0.000 | 0.000 |
| Moderate-stable, 3657 (45.8) | 0.007 (0.001, 0.013)^*^ | 0.004 (-0.002, 0.011) | 0.004 (-0.002, 0.011) |
| High-stable, 307 (3.9) | 0.001 (-0.014, 0.017) | 0.005 (-0.011, 0.021) | 0.005 (-0.011, 0.020) |
| Moderate-decreasing, 674 (8.6) | 0.031 (0.020, 0.041)^***^ | 0.034 (0.023, 0.045)^***^ | 0.034 (0.023, 0.046)^***^ |
| **IWRT** | | | |
| HDL-C, N (%) |  |  |  |
| Low-stable, 3926 (49.0) | 0.000 | 0.000 | 0.000 |
| Moderate-stable, 2960 (37.0) | 0.004 (-0.001, 0.010) | 0.004 (-0.002, 0.011) | 0.005 (-0.002, 0.011) |
| High-gradual, 839 (10.5) | 0.007 (-0.002, 0.016) | 0.005 (-0.005, 0.015) | 0.005 (-0.005, 0.015) |
| Moderate-increasing, 282 (3.5) | 0.019 (0.004, 0.034)^*^ | 0.019 (0.004, 0.034)^**^ | 0.021 (0.005, 0.036)^**^ |
| LDL-C, N (%) |  |  |  |
| Low-stable, 2900 (36.4) | 0.000 | 0.000 | 0.000 |
| Moderate-gradual, 4037 (50.6) | 0.005 (-0.001, 0.011) | 0.003 (-0.003, 0.010) | 0.003 (-0.003, 0.009) |
| High-gradual, 612 (7.7) | 0.008 (-0.002, 0.019) | 0.008 (-0.003, 0.020) | 0.008 (-0.003, 0.019) |
| High-decreasing, 419 (5.3) | 0.010 (-0002, 0.023) | 0.017 (0.003, 0.030)^*^ | 0.017 (0.003, 0.030)^*^ |
| TG, N (%) |  |  |  |
| Low-stable, 5804 (74.5) | 0.000 | 0.000 | 0.000 |
| Moderate-stable, 1614 (20.7) | -0.004 (-0.010, 0.003) | -0.003 (-0.010, 0.004) | -0.003 (-0.011, 0.004) |
| High-decreasing, 371 (4.8) | 0.006 (-0.007, 0.019) | 0.002 (-0.012, 0.016) | 0.002 (-0.012, 0.016) |
| TC, N (%) |  |  |  |
| Low-stable, 3319 (41.7) | 0.000 | 0.000 | 0.000 |
| Moderate-stable, 3657 (45.8) | 0.004 (-0.001, 0.010) | 0.003 (-0.003, 0.009) | 0.003 (-0.003, 0.009) |
| High-stable, 307 (3.9) | 0.001 (-0.013, 0.015) | 0.005 (-0.010, 0.020) | 0.005 (-0.010, 0.020) |
| Moderate-decreasing, 674 (8.6) | 0.007 (-0.003, 0.017) | 0.010 (0.001, 0.021)^*^ | 0.011 (0.001, 0.021)^*^ |
| **MMSE** | | | |
| HDL-C, N (%) |  |  |  |
| Low-stable, 1509 (47.9) | 0.000 | 0.000 | 0.000 |
| Moderate-stable, 1064 (33.8) | 0.006 (-0.002, 0.014) | 0.004 (-0.003, 0.011) | 0.005 (-0.003, 0.012) |
| High-gradual, 395 (12.6) | 0.001 (-0.011, 0.012) | 0.002 (-0.008, 0.013) | 0.004 (-0.006, 0.014) |
| Moderate-increasing, 179 (5.7) | 0.023 (0.007, 0.039)^**^ | 0.034 (0.021, 0.048)^***^ | 0.034 (0.020, 0.048)^***^ |
| LDL-C, N (%) |  |  |  |
| Low-stable, 1060 (33.7) | 0.000 | 0.000 | 0.000 |
| Moderate-gradual, 1533 (48.7) | -0.004 (-0.012, 0.004) | -0.004 (-0.011, 0.003) | -0.004 (-0.011, 0.003) |
| High-gradual, 329 (10.5) | -0.002 (-0.015, 0.010) | -0.003 (-0.013, 0.009) | -0.003 (-0.014, 0.008) |
| High-decreasing, 222 (7.1) | 0.009 (-0.005, 0.024) | 0.016 (0.003, 0.029)^*^ | 0.015 (0.002, 0.028)^*^ |
| TG, N (%) |  |  |  |
| Low-stable, 2327 (75.6) | 0.000 | 0.000 | 0.000 |
| Moderate-stable, 626 (20.3) | 0.001 (-0.008, 0.010) | 0.004 (-0.004, 0.012) | 0.003 (-0.005, 0.011) |
| High-decreasing, 127 (4.1) | -0.001 (-0.019, 0.018) | 0.003 (-0.013, 0.018) | 0.001 (-0.015, 0.017) |
| TC, N (%) |  |  |  |
| Low-stable, 1294 (41.1) | 0.000 | 0.000 | 0.000 |
| Moderate-stable, 1402 (44.6) | 0.001 (-0.007, 0.008) | 0.002 (-0.005, 0.009) | 0.002 (-0.005, 0.009) |
| High-stable, 126 (4.0) | 0.002 (-0.017, 0.021) | 0.003 (-0.013, 0.019) | 0.002 (-0.015, 0.017) |
| Moderate-decreasing, 325 (10.3) | 0.023 (-0.011, 0.035) | 0.028 (0.017, 0.038)^***^ | 0.027 (0.017, 0.038)^***^ |

DWRT = delayed 10-word recall test; IWRT = immediate 10-word recall test; MMSE = mini-mental state examination; HDL-C = high-density lipoprotein cholesterol; LDL-C = low-density lipoprotein cholesterol; TG = triglycerides; TC = total cholesterol; CI = confidence interval; N = number of participants

^a^: Model 1: adjusted for sex, age, baseline standardized IWRT/DWRT/MMSE scores, BMI, education, occupation, marital status, smoking status, drinking status, family income, physical activity, and self-rated health

^b^: Model 2: additionally adjusted for self-reported cardiovascular disease, hypertension, diabetes, hyperlipidemia and drug for hypertension, glucose or lipids

^*^: P < 0.05; ^**^: P < 0.01; ^***^: P < 0.001

**
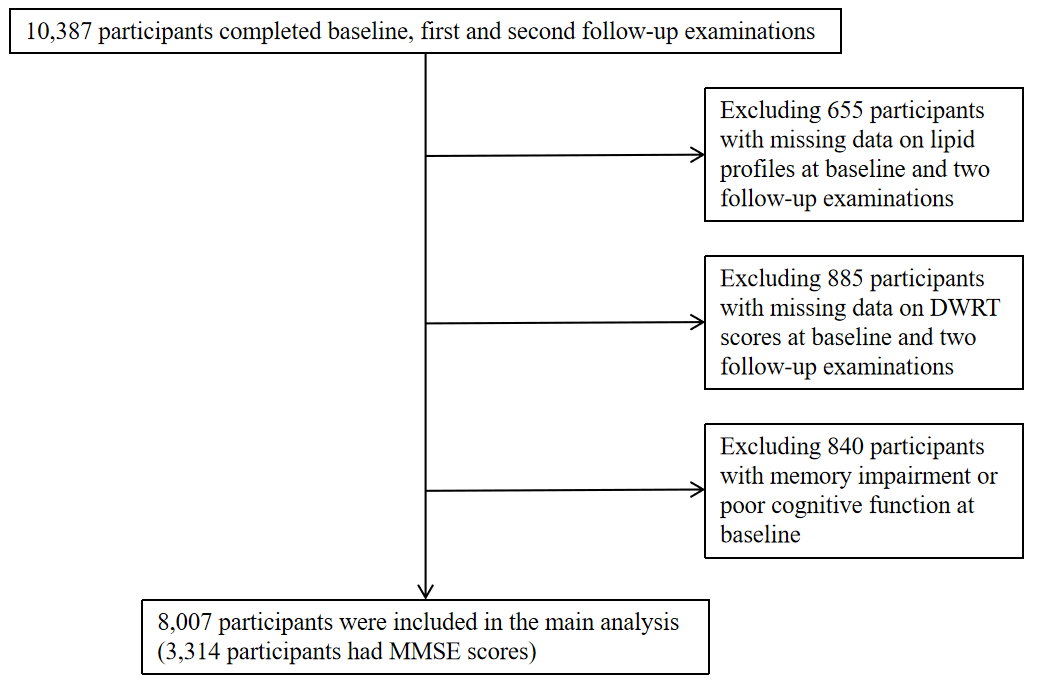
**

**Supplementary Figure 1.** The selection process of participants based on Guangzhou Biobank Cohort Study

Note: (1) Memory impairment was defined by DWRT scores < 4, and poor cognitive function was defined by MMSE scores < 25. (2) DWRT = delayed 10-word recall test; MMSE = mini-mental state examination.
